# CD24 regulates sorafenib resistance via activating autophagy in hepatocellular carcinoma

**DOI:** 10.1038/s41419-018-0681-z

**Published:** 2018-05-29

**Authors:** Shuai Lu, Yao Yao, Guolong Xu, Chao Zhou, Yuan Zhang, Jie Sun, Runqiu Jiang, Qing Shao, Yun Chen

**Affiliations:** 1 Department of Immunology, Nanjing Medical University, Nanjing, 211166 China; 2 Jiangsu Key Lab of Cancer Biomarkers, Prevention and Treatment, Collaborative Innovation Center for Cancer Personalized Medicine, Nanjing Medical University, Nanjing, 211166 China; 3 Key Laboratory of Human Functional Genomics of Jiangsu Province, Jiangsu Diabetes Center, Nanjing Medical University, Nanjing, 211166 China; 4 Department of Head and Neck Surgery, Cancer biotherapy Center, Jiangsu Cancer Hospital, The Affiliated Cancer Hospital of Nanjing Medical University, Nanjing, 210018 China; 5 Department of Ophthalmology, The First Affiliated Hospital of Nanjing Medical University, Nanjing, 210029 China; 6 Liver Transplantation Center, The First Affiliated Hospital of Nanjing Medical University, Nanjing, 210029 China

## Abstract

Hepatocellular carcinoma is one of most common solid cancers worldwide. Sorafenib is indicated as a treatment for advanced hepatocellular carcinoma (HCC). However, the clinical efficacy of sorafenib has been severely compromised by the development of drug resistance, and the precise mechanisms of drug resistance remain largely unknown. Here we found that a cell surface molecule, CD24, is overexpressed in tumor tissues and sorafenib-resistant hepatocellular carcinoma cell lines. Moreover, there is a positive correlation between CD24 expression levels and sorafenib resistance. In sorafenib-resistant HCC cell lines, depletion of CD24 caused a notable increase of sorafenib sensitivity. In addition, we found that CD24-related sorafenib resistance was accompanied by the activation of autophagy and can be blocked by the inhibition of autophagy using either pharmacological inhibitors or essential autophagy gene knockdown. In further research, we found that CD24 overexpression also leads to an increase in PP2A protein production and induces the deactivation of the mTOR/AKT pathway, which enhances the level of autophagy. These results demonstrate that CD24 regulates sorafenib resistance via activating autophagy in HCC. This is the first report to describe the relationships among CD24, autophagy, and sorafenib resistance. In conclusion, the combination of autophagy modulation and CD24 targeted therapy is a promising therapeutic strategy in the treatment of HCC.

## Introduction

Hepatocellular carcinoma (HCC) is the most common type of primary liver cancer in the world^1^. As with any other cancer, the treatment and prognosis of HCC vary depending on the specifics of tumor pathology, size and the overall health of the patient^2^. Most HCC patients are diagnosed in advanced stages, and thus there is an urgent need for novel treatments for advanced HCC^3,4^. Sorafenib, a small inhibitor of several tyrosine-protein kinases, has been shown to be effective in patients with advanced HCC^5,6^. The effects of sorafenib include blocking the Raf-MEK-ERK signaling pathway to inhibit tumor cell proliferation and target the vascular endothelial growth factor receptor (VEGFR) and platelet derived growth factor receptor (PDGFR) to prevent angiogenesis^7^. Despite this encouraging advance, drug resistance to sorafenib remains a serious concern as the overall survival (OS) of HCC patients after sorafenib treatment is only 2–3 months longer than placebo^8,9^.There are three main reasons for sorafenib resistance in hepatocellular carcinoma: First, abnormal changes in vascular endothelial growth factor receptor (VEGFR) and its downstream signaling pathway^10^; second, overexpression of silent information regulator 1 (SIRT1)-induced sorafenib resistance^11^; and third, activation of autophagy, which may enhance sorafenib resistance in hepatocellular carcinoma^12^. However, there are still many other mechanisms which may contribute to sorafenib resistance. In this study, we elucidated a new mechanism of resistance.

CD24 is a glycoprotein expressing on the surface of most B lymphocytes^13^ and several tumor types, including prostate cancer^14^, cervical cancer^15^, non-small cell lung carcinoma^16^, gastric cancer^17^, and breast cancer^18^. The encoded protein is anchored via a glycosyl phosphatidylinositol (GPI) linked to the cell surface and contributes to a wide range of downstream signaling networks^13^. The depletion of CD24 caused a notable decrease in cell proliferation, migration, and invasion in vitro^19^. In our study, we confirmed that CD24 is highly expressed in HCC tumor tissues compared to the adjacent tissues. Interestingly, the expression of CD24 increased significantly in residual chemoresistant patients upon sorafenib treatment when compared to the untreated patients, suggesting that CD24 participates in a sorafenib-induced resistance process. However, there is no report on the role of CD24 in sorafenib resistance. Hence, we studied the relationship between CD24 and sorafenib resistance in hepatocellular carcinoma.

Through clinical sampling, we also found that CD24 overexpression in patients was accompanied by the activation of autophagy^17^. Autophagy allows the orderly degradation and recycling of cellular components^20,21^. The role of autophagy in cancer is one that has been highly researched in recent years. More and more evidence points to the role of autophagy both as a tumor suppressor and as a factor in tumor cells^22–24^. In a recent study, several articles reported that heat shock factor protein1 (HSF1)^25,26^ and reactive oxygen species (ROS)^27^-mediated autophagy activation advance drug resistance in tumor cells. However, both how CD24 overexpression induces autophagy and whether autophagy activation contributes to tumor cell drug resistance or is a mechanism of resistance remain uncertain. Therefore, we hypothesized that CD24 regulates sorafenib resistance via activating autophagy in HCC.

The phosphatidylinositol 3-kinase/Akt/mammalian target of rapamycin (PI3K/Akt/mTOR) pathway is a key regulator of autophagy^28,29^. From a whole-transcriptome shotgun sequencing (RNA-Seq) study, we determined the segment responsible for the downregulation of the mTOR/AKT pathway in sorafenib resistance cells. In addition, this pathway was defective in sorafenib-resistant cells in a dose-dependent manner and was rescued by CD24 knockdown, suggesting that CD24-induced autophagy activation through the inhibition of the mTOR/AKT pathway. MTOR is mainly composed of mTOR complex 1 (mTORC1) and mTOR complex 2 (mTORC2), which differ in their subcellular localization and binding partners^30^. Whereas mTORC1 regulates the autophagy activation phosphorylation/dephosphorylation of autophagy-related gene 13 (Atg13) directly and regulates translation through 4EBP1 and p70S6K, mTORC2 activates AKT through phosphorylation at Ser473^31–33^. Protein phosphatase 2 (PP2A)^34^, a multifunctional enzyme that inhibits AKT activity, was significantly increased in CD24-overexpressing cells from this study. Therefore, PP2A may play an important role in CD24-mediated autophagy activation.

Hence, in this study, we sought to identify the relationship among CD24, autophagy, and sorafenib resistance. Targeting this relationship, which we hypothesize is a novel mechanism of drug resistance, may have therapeutic implications for the treatment of HCC.

## Results

### High expression of CD24 in human HCC is associated with sorafenib resistance

**Fig. 1:**
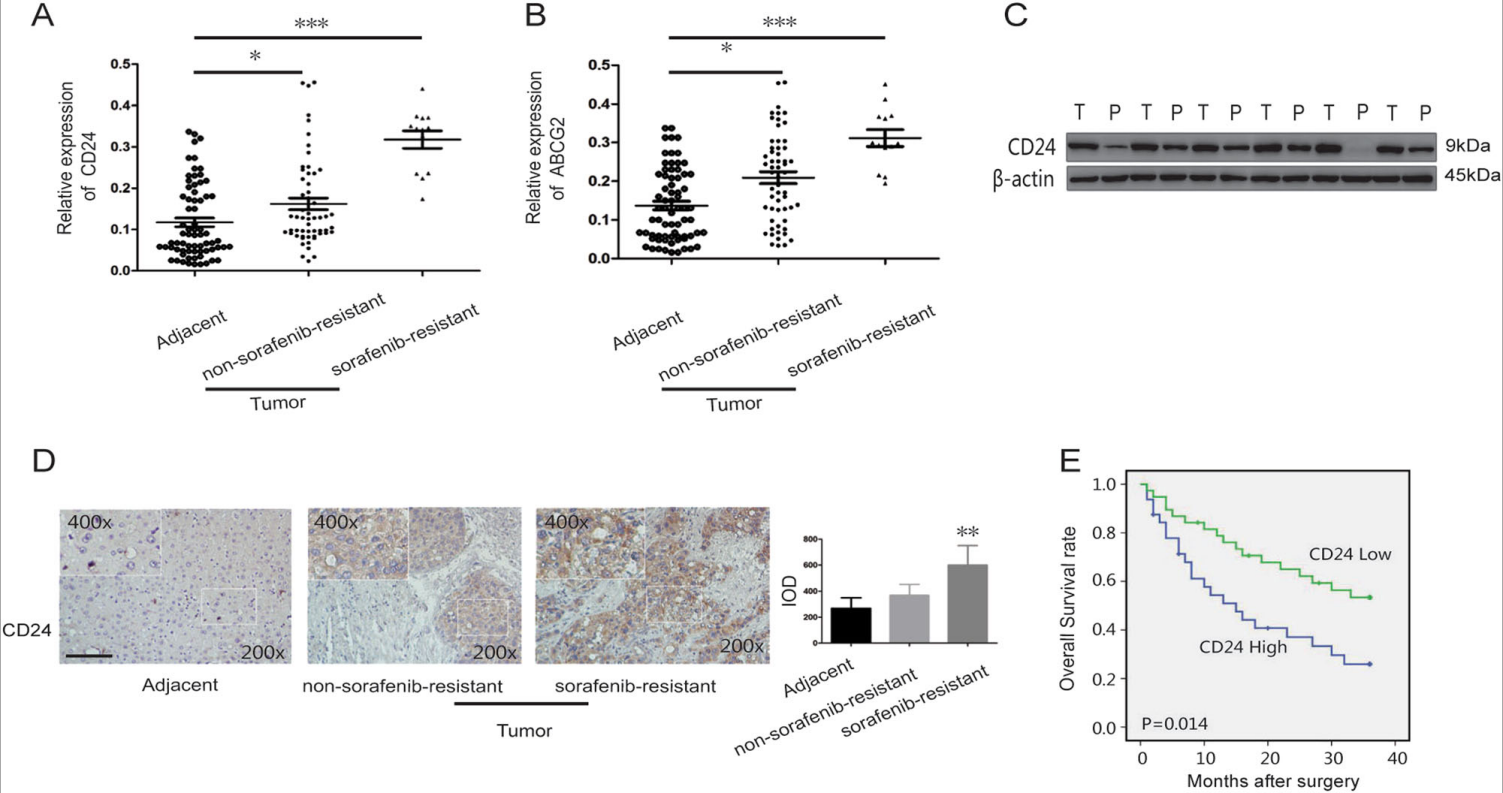
CD24 expression is higher in tumor tissue samples of patients with poor prognosis. **a**, **b** Scatter plot of the CD24 and ABCG2 mRNA expression in tumor tissues and the corresponding adjacent tissues of HCC patients (*n* = 70). Patients with sorafenib resistance (13 of 70) had high expression of CD24. **c** The relative expression level of the CD24 protein in tumor tissues(T) and the corresponding adjacent tissues(P) of HCC patients. **d** The positive percent of CD24 was detected by IHC staining in 16 paraffin-embedded HCC and adjacent tissue specimens. The original magnification was ×200. **e** Patients with positive CD24 expression presented a poor overall survival (OS) compared with the negative group. Seventy patients were divided into the CD24 positive group (*n* = 32) and CD24 negative group (*n* = 38).Representative images are shown on the left, and quantitative data are on the right. Each experiment was performed in triplicate. Data are presented as the means ± s.e.m. and analyzed with the Student’s *t*-test (*P < 0.05, **P < 0.01)

To determine whether CD24 marks sorafenib-resistant HCC cells, we examined its expression by qRT-PCR in the tumor and the corresponding adjacent tissues of HCC patients (*n* = 70). First, we found that the expression of CD24 was higher in the tumor tissues than adjacent tissues; moreover, the clinically diagnosed sorafenib-resistant tumor tissues (*n* = 13) expressed higher levels of CD24 compared to the non-sorafenib-resistant tissues (*n* = 57) (Fig. 1a). Next, we detected the transcription of a sorafenib-resistance associated protein, ATP-binding cassette sub-family G member 2 (ABCG2)^9^. We found that the tumor cells of HCC patients have significantly higher ABCG2 than the adjacent normal liver tissues, while the cells of sorafenib-resistant HCC patients expressed the highest ABCG2 (Fig. 1b). The protein expression of CD24 was verified by both western blot and IHC staining, and the results indicated that CD24 was highly expressed in human HCC tissues especially in tumor cells, and that sorafenib-resistant cells expressed significantly higher levels of CD24 (Fig. 1c, d). Furthermore, the survival analysis showed that patients with high-CD24 expression in HCC had significantly worse prognosis than those with low-CD24 expression (*p* = 0.014) (Fig. 1e).

To further study the biological effects, as well as molecular mechanisms of CD24 associated sorafenib-resistance, we established two HCC cell lines by long-term exposure to sorafenib at low doses (0.625 μM) escalating to higher doses, up to the cell surviving in medium with 10 μM (the highest clinically achievable concentration) sorafenib concentration (Supplementary Figure 1A). We obtained Huh7 and Hep3B sorafenib-resistant cells (Huh7/SR and Hep3B/SR). We measured sorafenib sensitivity by CCK8 assay (48 h), and the result indicated that sorafenib-resistant cells possess lower sorafenib sensitivity (Supplementary Figure 1B). Drug resistance-association proteins (ABCG2) were highly expressed in sorafenib-resistant cells by western blot assays (Supplementary Figure 1C). In addition, sorafenib-resistant cells exhibited higher cell proliferation and lower apoptosis in the presence of sorafenib compared to wild-type cells (Supplementary Figure 1D,E). These results suggested that we successfully established sorafenib-resistant HCC cell line models.

**Fig. 2:**
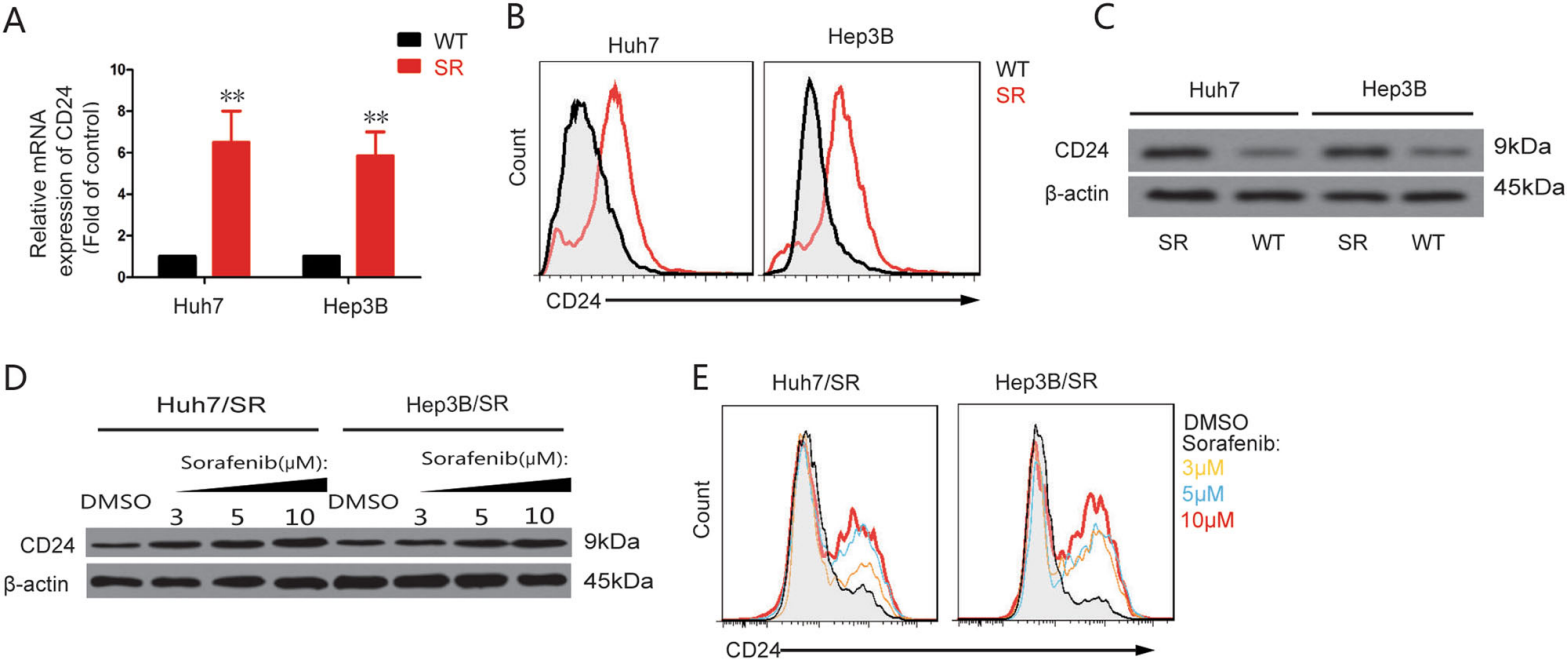
CD24 expression is higher in sorafenib-resistant HCC cells. CD24 expression was higher in Huh7/SR and Hep3B/SR cells that were assayed by (**a**) qRT-PCR, (**b**) Flow cytometer, and (**c**) western blot. In addition, western blot (**d**) and the flow cytometer assays (**e**) indicated the expression of CD24 in a dose-dependent manner in the presence of sorafenib. SR sorafenib-resistant cell. WT wild-type cell. Each experiment was performed in triplicate. The data are presented as the means ± s.e.m. and analyzed with Student’s *t*-test (*P < 0.05, **P < 0.01)

We further detected CD24 expression in the sorafenib-resistant cells. A significant increase of CD24 was detected in sorafenib-resistant cells by using qRT-PCR, flow cytometry, as well as western blot (Fig. 2a–c). In addition, the presence of sorafenib affected the expression of CD24 in sorafenib-resistant cells in a dose-dependent manner (Fig. 2d, e). Hence, CD24 was overexpressed in sorafenib-resistant cells with higher cell proliferation and lower apoptosis in the presence of sorafenib.

**Fig. 3:**
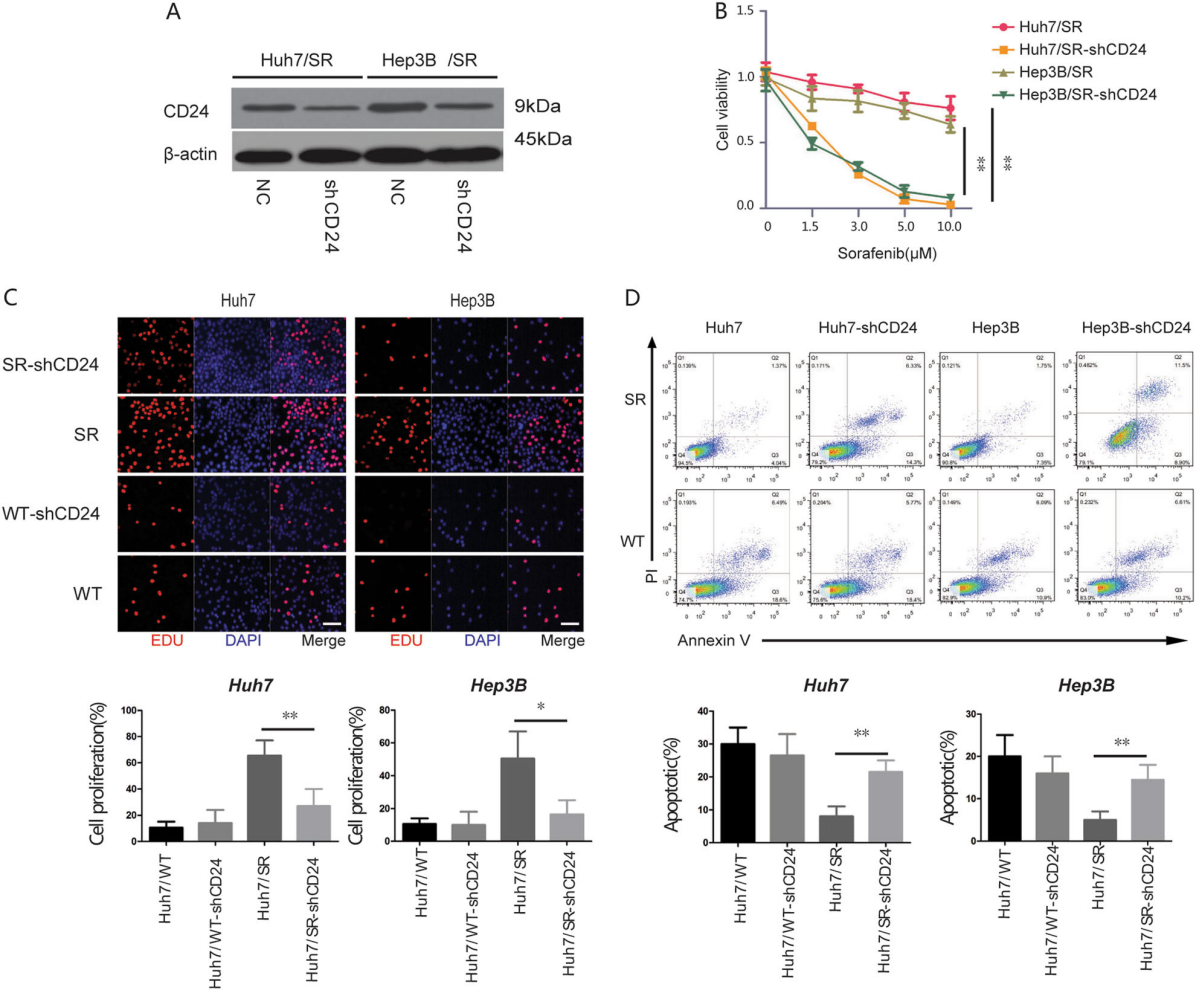
CD24 Knockdown reduced sorafenib resistance. **a** Western blot was used to assay the knockout efficiency of CD24. **b** Huh7/SR and Hep3B/SR cells were treated with different concentrations (0, 1.5, 3.0, 5.0, 10.0 μM) of sorafenib. After 48 h, the CCK8 assay was used to detect sorafenib sensitivity in the cells. Cells were treated with 1.5 μM sorafenib for 48 h. **c** Cell proliferation detected by EDU assays. The original magnification was ×400. **d** Apoptosis detected by AnnexinV-PI assays. Representative images are shown on the top, and quantitative data are on the bottom. Each experiment was performed in triplicate. Data are presented as the means ± s.e.m. and analyzed with Student’s *t*-test (*P < 0.05, **P < 0.01)

To better understand whether CD24 was necessary for sorafenib resistance, we knocked-down CD24 with a small hairpin RNA (shCD24) in Huh7/SR and Hep3B/SR cells. Knockdown efficiency was verified by western blot and qRT-PCR (Fig. 3a and supplementary figure 2). We investigated several malignancy associated factors. First, the cell viability was detected by CCK8 assay 48 h after the treatment of various concentration of sorafenib, and we found that downregulation of CD24 can decrease the cell viability of Huh7/SR and Hep3B/SR cells especially with the treatment of sorafenib over 1.5 μM (Fig. 3b). Also, knockdown of CD24 in Huh7/SR and Hep3B/SR significantly decreased their malignant behaviors including proliferation (Fig. 3c), and anti-apoptosis ability with the treatment of sorafenib (1.5 μM) (Fig. 3d). Therefore, we concluded that CD24 was associated with sorafenib resistance. However, the underlying molecular mechanism should be further explored.

### CD24 associated sorafenib resistance is mediated by PP2A/AKT/mTOR signaling

**Fig. 4:**
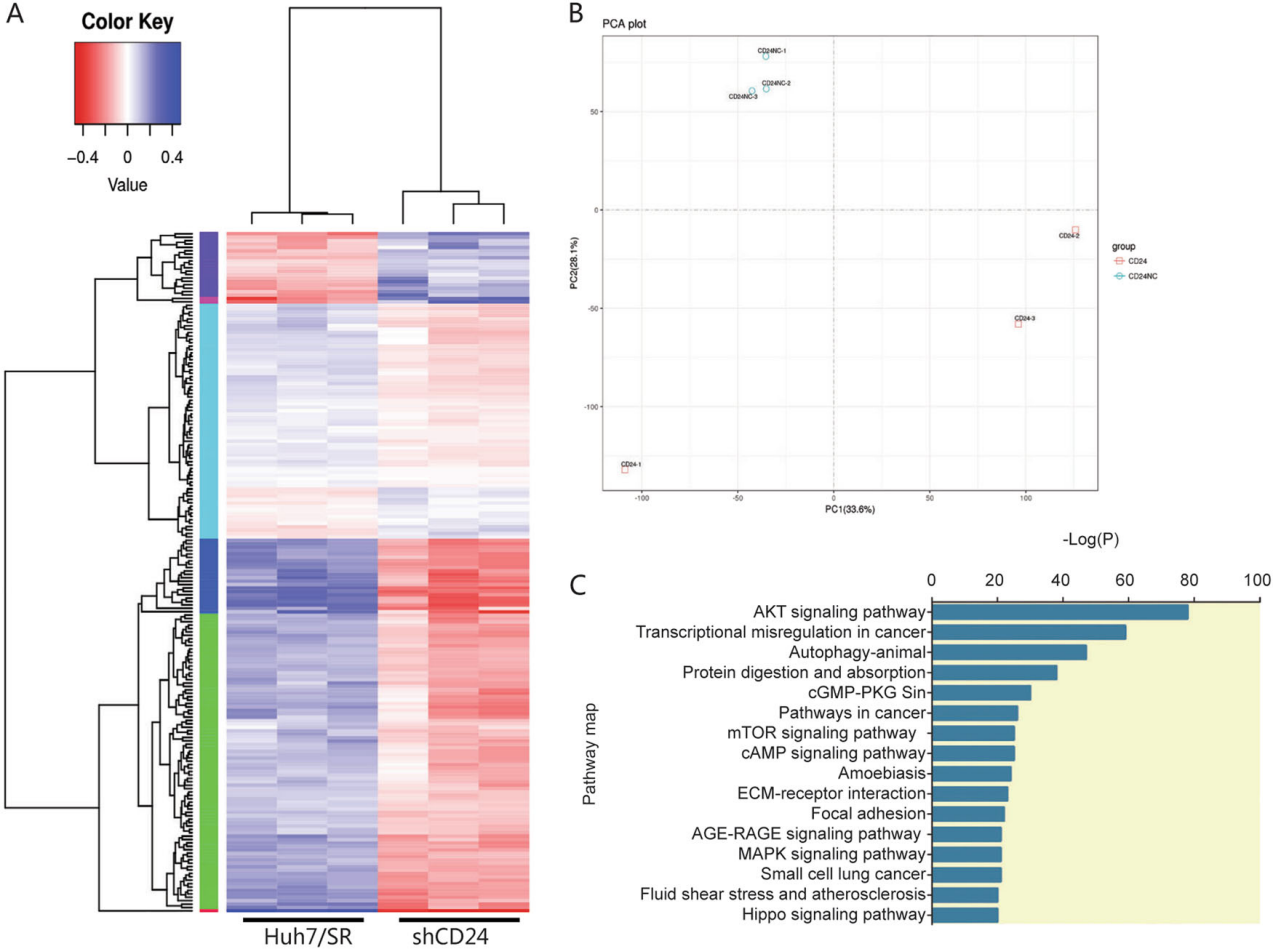
Landscape of mRNA expression in CD24 knockdown Huh7/SR and normal control Huh7/SR cells. **a** Hierarchical clustering analysis of 200 mRNAs that are differentially expressed (the threshold of significance was defined as *P* < 0.05 (with Student’s *t*-test) and the false discovery rate) in CD24 knockdown Huh7/SR cells (shCD24), and normal control Huh7/SR cells. The clustering tree for the mRNAs is shown at the top. The expression values are shown in shades of blue and red, indicating expression above and below the median expression value across all the samples (log scale 10, from –0.4 to +0.4), respectively. **b** A principal component analysis (PCA) revealed that the samples derived from the two groups displayed a tendency to form separate clusters when analyzed for mRNAs. The position of each dot represents the value of the sample on each principal component. **c** Pathway analysis showing the significant pathways of the differentially expressed protein-coding genes (*P* < 0.05 with Student’s *t*-test)

To understand the mechanism of CD24-induced sorafenib-resistance, we carried out a whole-transcriptome shotgun sequencing (RNA-Seq) to determine the continually cellular transcriptome change between Huh7/SR and Huh7/SR shCD24 (treatment with 1.5 μM sorafenib). In an unsupervised clustering analysis of all the transcripts, we detected significant differences in the expression signatures of the two sets of samples (*n* = 3 for each) (Fig. 4a). A principal component analysis (PCA) revealed that the samples derived from the two groups displayed a tendency to form separate clusters when analyzed for mRNAs (Fig. 4b). Using the differentially expressed mRNAs as the input, we analyzed the significant pathways associated with them using the Kyoto Encyclopedia of Genes and Genomes (KEGG), BioCarta and Reactome Pathway Database platforms. We identified many enriched pathways, among which the AKT/mTOR and autophagy pathways were the most significant (Fig. 4c) but were further investigated elsewhere.

**Fig. 5:**
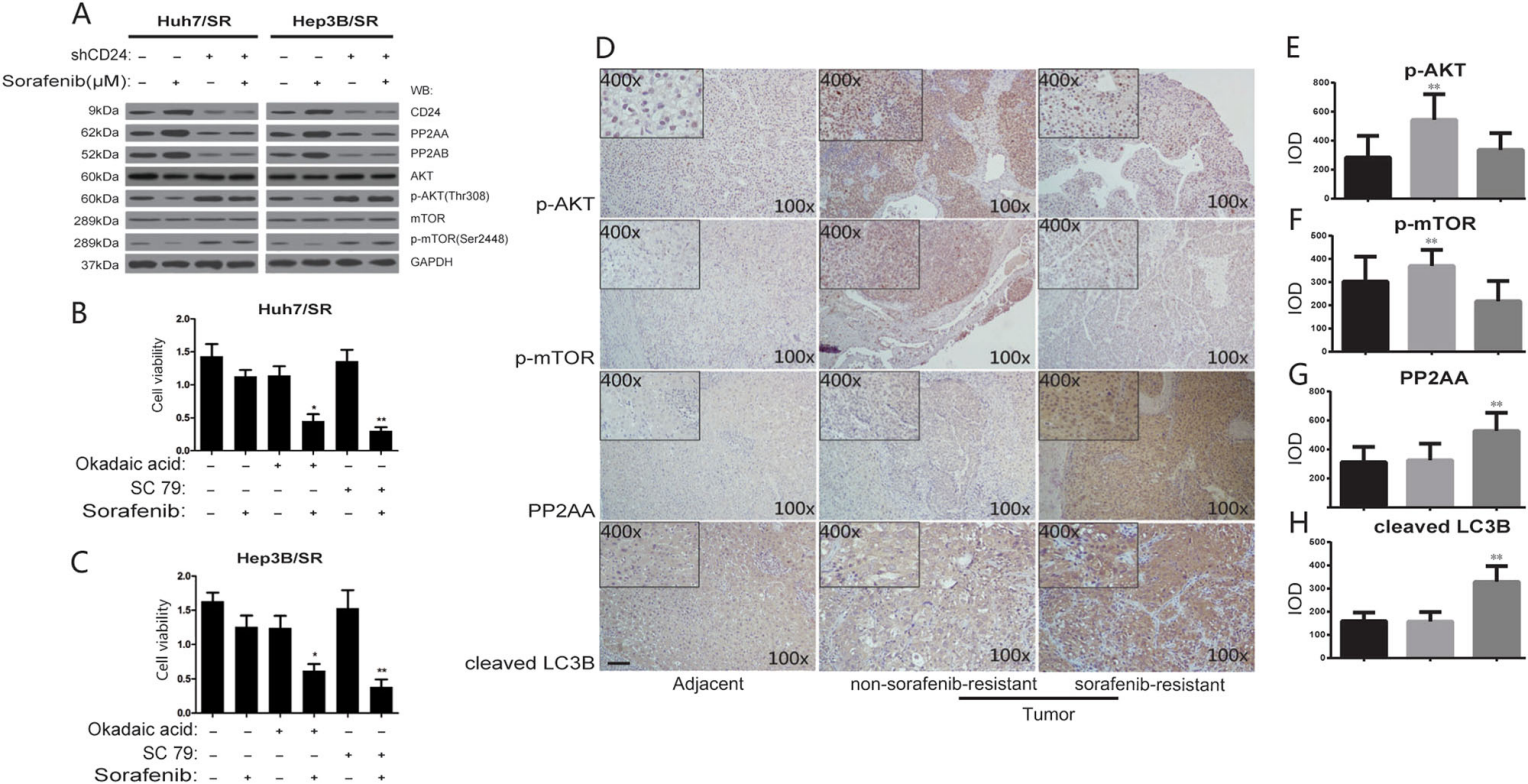
CD24 associated sorafenib resistance is mediated by PP2A/AKT/mTOR signaling. CD24 knockdown (shCD24) and normal control Huh7/SR, Hep3B/SR cells were cultured with 1.5 μM sorafenib for 48 h, then subjected to western blot analysis. **a** The ratio of phospho-mTOR (Ser2448) and phospho-AKT (Thr308) was decreased in cells, and the expression of PP2A was dramatically increased. **b** Huh7/SR and **c** Hep3B/SR cells were treated with the PP2A inhibitor Okadaic acid (1 nM) and AKT agonist, SC79 (1 nM). After 48 h, the CCK8 assay was used to detect cell viability. IHC staining (**d**) and integrated optical density (IOD) was evaluated for (**e**) p-AKT(Thr308), (**f**) p-mTOR, (**g**) PP2AA, and (**h**) cleaved LC3B in human HCC adjacent tissues and non-sorafenib-resistant and resistant tissue. The original magnification was ×100. Each experiment was performed in triplicate. The data are presented as the means ± s.e.m. and analyzed with Student’s *t*-test (*P < 0.05, **P < 0.01)

To confirm the prediction of the RNA-Seq, we detected the activation of AKT/mTOR signaling with the treatment of sorafenib (1.5 μM). We found that the phosphorylation of AKT (p-AKT) in Thr308, as well as p-mTOR (Ser2448) decreased dramatically in Huh7/SR cells but that there was no difference in Huh7/SR shCD24 cells. The same results were found in Hep3B/SR cells. These results might indicate that CD24 regulates sorafenib resistance by inhibiting the activation of AKT/mTOR signaling. We also found that one of the inhibitors of AKT, PP2A was dramatically increased through the RNA-seq data, and we further confirmed its transcription and protein expression (Fig. 5a). We postulated that CD24 was associated with sorafenib resistance through PP2A induced AKT/mTOR suppression. We investigated the cell viability change with the treatment of the PP2A inhibitor Okadaic acid and AKT agonist, SC79. There was no significant cell viability change without sorafenib treatment (1.5 μM), but the cell viability decreased significantly when either PP2A was inhibited or the activity of AKT was boosted in both Huh7/SR and Hep3B/SR (Fig. 5b, c). We further analyzed the activation of AKT/mTOR with the treatment indicated above. We found that the decreased cell viability was associated with activation of AKT/mTOR signaling with the treatment of sorafenib. Since the RNA-Seq data also indicated a significant difference in autophagy, and autophagy is one of the downstream effects of AKT/mTOR signaling, we continued to explore the roles of autophagy in CD24 mediated sorafenib resistance. Based on this hypothesis, we investigated such signaling in human clinical samples by using IHC staining, and found that compared to the sorafenib-sensitive tissues, sorafenib-resistant HCC tissues showed significantly lower activation of AKT/mTOR, but increased expression of PP2A and LC3-II (Fig. 5d–h). Therefore, we focused on the roles of CD24 mediated autophagy in sorafenib resistance.

### Autophagy plays an essential role in CD24 associated sorafenib resistance

**Fig. 6:**
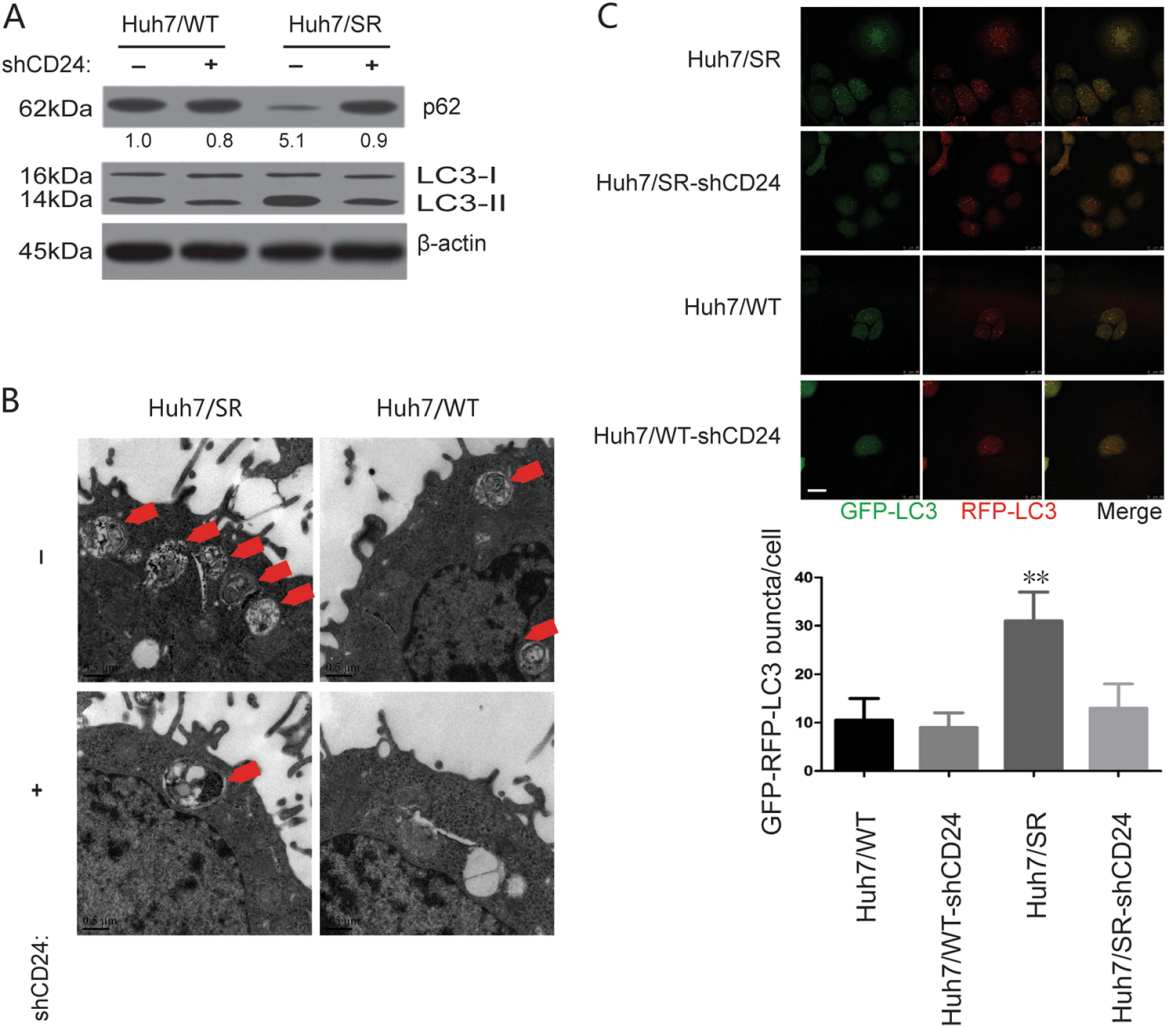
CD24 overexpression regulates autophagy activation in sorafenib resistance. The level of autophagy was higher in Huh7/SR than Huh7/WT cells, but not in CD24 knockdown cells. **a** The relative expression level of LC3B and p62 protein was analyzed by western blot. The relative amounts of LC3-II are indicated. **b** Evaluation of autophagic vacuoles (AVs) by transmission electron microscopy. Cells were cultured with 1.5 μM sorafenib for 48 h then subjected to transmission electron microscopy analysis. The area noted by the red arrows is the autophagic vacuole (AV). Scale bars, 0.5 μm. **c** The GFP and RFP signals of tandem fluorescent LC3 (RFP-GFP-LC3) show different localization patterns. Cells were transfected with plasmids expressing either tandem fluorescent LC3. Twenty-four hours after transfection, the cells were starved in Hanks balanced salt solution for 2 h, fixed and analyzed by microscopy. Representative images are shown on the left, and quantitative data are on the right. Scale bars, 5 μm. Bar diagram (mean ± s.e.m.) representing the average “yellow only” spot counts/cell (*P* < 0.05 with Student's *t*-test). Each experiment was performed in triplicate. The data are presented as the means ± s.e.m. and analyzed with Student’s *t*-test (*P < 0.05, **P < 0.01)

We first examined the levels of autophagy in Huh7/SR cells and Huh7/SR CD24shRNA. We found increased LC3-II but decreased p62 in Huh7/SR cells compared to Huh7/SR CD24shRNA with the treatment of 1.5 μM sorafenib (24 h). (Fig. 6a). Transmission electron microscopy showed that a large number of double-membrane vacuolar structures that are morphological features of autophagosomes existed in Huh7/SR cells, but not in the Huh7/SR CD24shRNA cells (Fig. 6b). A fluorescence assay is designed to monitor the autophagosomes by using RFP-GFP-tagged LC3. The GFP signal is sensitive to the acidic and/or proteolytic conditions, whereas RFP is more stable. The fusing signaling (yellow dots) indicates that a compartment has not fused with a lysosome, such as the phagophore or an autophagosome. With this standard, we found that Huh7/SR cells had stronger autophagy compared to Huh7/SR shCD24 cells (Fig. 6c).

**Fig. 7:**
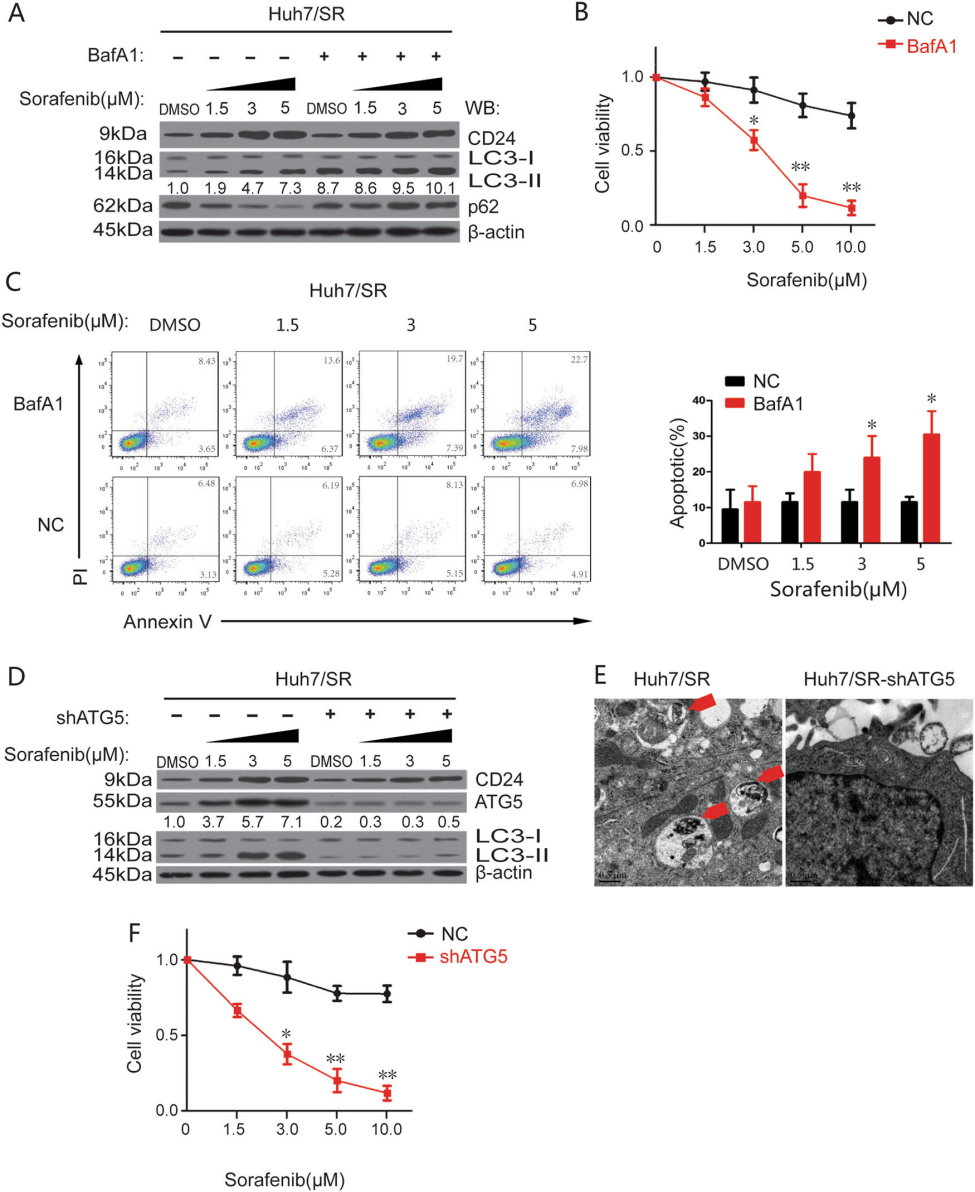
Autophagy was necessary for CD24-induced sorafenib resistance progress. The effects of CD24-dependent sorafenib resistance will soon disappear with the inhibition of autophagy using either pharmacological inhibitors or essential autophagy gene knockdown. We treated Huh7/SR cells with 1 nM bafiIomycinA1 (BafA1) to inhibit autophagy by blocking AV-lysosome fusion. Cells were treated with different concentrations (0, 1.5, 3, 5, 10.0 μM) of sorafenib for 48 h. **a** The relative expression level of LC3B, p62, and CD24 protein was analyzed by western blot. The relative amounts of LC3-II are indicated. **b** CCK8 assay was used to detect cell sorafenib sensitivity. **c** Apoptosis detected by AnnexinV-PI assays. Representative images are shown on the left, and quantitative data are on the right. We knocked-down ATG5 with a small hairpin RNA (shATG5) in Huh7/SR cells. **d** The relative expression level of ATG5, LC3B, and CD24 protein was analyzed by western blot. The relative amounts of LC3-II are indicated. **e** Evaluation of the AVs by transmission electron microscopy. Cells were cultured with 1.5 μM sorafenib for 48 h then subjected to transmission electron microscopy analysis. The area noted by red arrows is the autophagic vacuole (AV). Scale bars, 0.5 μm. **f** CCK8 was used to detect the cell sorafenib sensitivity. Each experiment was performed in triplicate. The relative expression level of LC3B, ATG5, and CD24 protein was analyzed by western blot. The relative amounts of LC3-II are indicated. Each experiment was performed in triplicate. The data are presented as the means ± s.e.m. and analyzed with Student’s *t*-test (*P < 0.05, **P < 0.01)

To investigate whether autophagy was necessary for CD24-induced sorafenib resistance progression, we treated Huh7/SR cells with 1 nM bafiIomycinA1 (BafA1), which inhibits autophagy by blocking autophagic vacuole (AV)-lysosome fusion. Although there were no obvious changes in CD24, the autophagy in Huh7/SR cells was significantly inhibited (Fig. 7a), resulting in an increased sensitivity to sorafenib compared to the BafA1-untreated cells (Fig. 7b). We found this sorafenib sensitivity was due to increased apoptosis (Fig. 7c). We also blocked autophagy by knocking-down ATG5, the key protein involved in the extension of the phagophoric membrane in autophagic vesicles^35^, and autophagy was significantly blocked in Huh7/SR shATG5 cells by both western blot and transmission electron microscopy (Fig. 7d, e). Similarly to the BafA1 treatment, sorafenib sensitivity increased significantly in Huh7/SR shATG5 cells (Fig. 7f). Therefore, we concluded here that autophagy plays an essential role in CD24 associated sorafenib resistance.

### CD24 enhanced tumor sorafenib resistance by increase autophagy in vivo

**Fig. 8:**
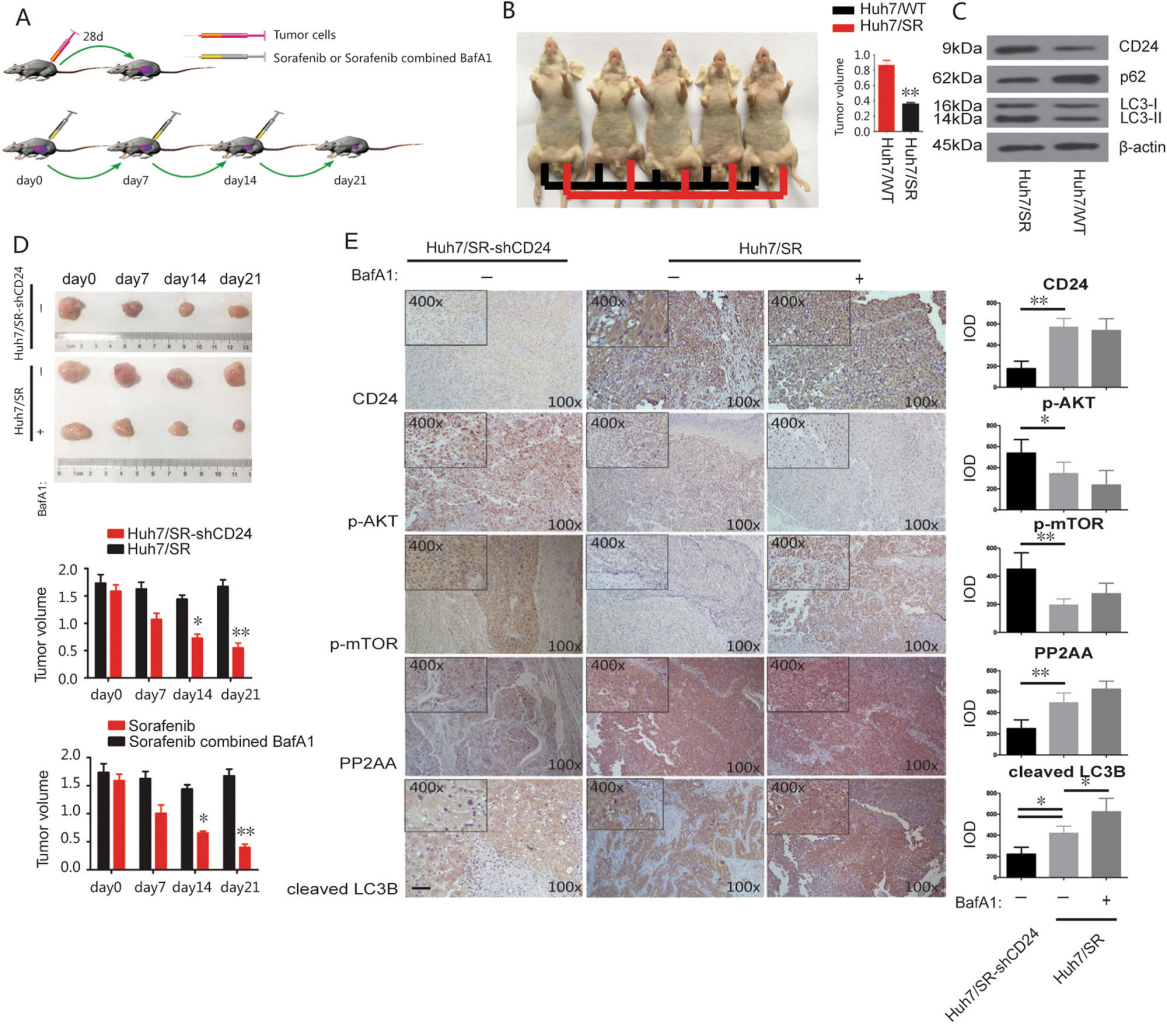
The in vivo relationship between CD24, autophagy, and sorafenib resistance. **a** The protocol of the experiment is shown in the diagram. **b** There are ~5 × 10^6^ Huh7/WT and Huh7/SR cells that were injected into the left and right flank of the mice. Once tumors reached a size of ≥200 mm^3^, the mice were treated with 100 mg/kg sorafenib diluted in sterile PBS by injection once a week. Tumor diameters were serially measured with calipers, and tumor volumes were calculated as: volume = width^2^ × length/2. **c** Tumor cells separated from the flank of mice were examined for the relative expression level of LC3B, P62, and CD24 protein by the western blot. **d** Huh7/SR and Huh7/SR shCD24 cells were injected into the flank of the mice. As stated before, mice were treated with 100 mg/kg sorafenib and 20 mg/kg BafA1 in sterile PBS by injection once a week. The tumor volumes were measured by calipers. Representative images are shown on the top, and quantitative data are on the bottom. **e** IHC staining for CD24, p-AKT, p-mTOR, PP2A-alpha, and cleaved LC3 in the tumor tissues from different mice models indicated in the figures. Representative images are shown on the left, and quantitative data are on the right. The original magnification was ×100. Each experiment was performed in triplicate. The data are presented as the means ± s.e.m. and analyzed with Student’s *t*-test (*P < 0.05, **P < 0.01)

As shown in the diagram (Fig. 8a), ~5 × 10^6^ Huh7/WT and Huh7/SR cells were injected into the left and right flank of mice respectively. Once tumors reached a size of ≥200 mm^3^, the mice were treated with 100 mg/kg sorafenib diluted in sterile PBS by injection once a week. After 3 weeks, we found that the Huh7/SR derived tumor grew faster than the Huh7/WT tumor (Fig. 8b). We found increased expression of CD24 in the tumor tissues by western blot, indicating that CD24 might be associated with sorafenib resistance in vivo (Fig. 8c). To further study, the relationship between CD24-induced sorafenib resistance and autophagy, we performed in vivo tumorigenesis with Huh7/SR and Huh7/SR shCD24 cells. After 3 weeks, the tumors were treated with sorafenib and BafA1 (20 mg/kg), and tumor volume was monitored during the treatment. We found that sorafenib inhibited the tumor growth derived from Huh7/SR shCD24 but had only a slight effect on the Huh7/SR tumor. However, when sorafenib treatment was combined with BafA1, an autophagy inhibitor, the tumor growth of Huh7/SR was dramatically inhibited (Fig. 8d). This result indicated that CD24-dependent sorafenib resistance was mainly determined by autophagy activation in vivo. Finally, we detected the protein expression of CD24, p-AKT, p-mTOR, PP2A-alpha, and cleaved LC3 in the tumor tissues by using IHC staining, and the results indicated that the treatment of CD24shRNA can block CD24-induced autophagy via activation of AKT/mTOR signaling through decreasing the expression of PP2A alpha. The treatment of BafA1 had no effect on the expression and activation of AKT/mTOR or autophagy (Fig. 8e).

## Discussion

CD24 is a glycosylphosphatidylinositol-anchored membrane protein that functions as an adhesion molecule for P-selectin and L1 and plays a role in cell selection and maturation during hematopoiesis, B-cell development, and neurogenesis^36,37^. More recently, studies have implicated CD24 expression in many types of tumorigenesis and progression^15–18,36^. However, in different tumors or changing tumor incidence environments, there are various effects of CD24 and diverse activation of its downstream signals^38,39^. Recently, research based on transcript profiling indicates that CD24 is highly expressed in HCC and might be a good biomarker for the prediction of HCC prognosis^40^. This conclusion was also reached by transcript profile analysis in our institute^19^. Until now, systematic investigation of the prognostic significance of CD24 in HCC has not been reported, especially in long-term studies or large numbers of patients at follow-up. Moreover, the role of CD24 in HCC progression has not been clearly defined. In our study, we found that CD24 is highly expressed in HCC tumor tissues compared to adjacent tissues^19^. It is noteworthy that the expression of CD24 significantly increased in residual chemoresistant patients upon sorafenib treatment when compared with untreated patients. Some articles have reported that CD24-mediated amplification of the Met cascade may contribute to the drug resistance of endometrial cancer^41^. In vitro, we detected a positive correlation between CD24 and sorafenib resistance in a dose-dependent manner. Currently, sorafenib has been applied as a first-line systemic therapy for advanced HCC^6^. However, the direct mechanism for tumor lethality mediated by sorafenib remains to be fully characterized, especially in HCC.

This study first demonstrated the association of CD24 with sorafenib resistance in HCC. Previous studies have shown that CD24 is mostly associated with drug resistance as a cancer stem cell marker molecule^42–44^. To investigate the relationship between CD24 and sorafenib resistance in vitro, we successfully established a sorafenib-resistant HCC cell line model. Sorafenib-resistant cells exhibit higher levels of cell proliferation, invasion, and lower apoptosis in the presence of sorafenib compared with WT cells. In this model, we have confirmed that CD24 was overexpressed in sorafenib-resistant cells. To explore the significant role of CD24 in sorafenib resistance, we knocked-down CD24 with a small hairpin RNA (shCD24) in sorafenib-resistant cells. We observed the level of sorafenib sensitivity, which was increased compared to Huh7/SR. Hence, overexpression of CD24 results in sorafenib resistance progression in HCC.

According to the analysis of transcriptome shotgun sequencing (RNA-Seq), we focused on the following annotated pathways, including the AKT/mTOR signaling pathways and autophagy pathways. Many studies have clearly indicated that autophagy is a cytoprotective mechanism mediating drug-resistance^45,46^. In our present study, we confirmed high levels of autophagy in patient HCC samples. Sorafenib treatment in sorafenib-resistant cells induced the morphological and biochemical hallmarks of autophagy, such as the generation of autophagosomes, RFP-GFP-LC3 redistribution and LC3-II accumulation. LC3 is widely used as a marker for the microscopic detection of isolation membranes and autophagosomes^47^. In addition, PE-conjugated LC3 (LC3-II) and unconjugated LC3 (LC3-I) can be detected separately by immunoblot analysis, and the amount of LC3-II is also widely used for the quantification of autophagic activity. It has been suggested that LC3 functions in the closure of the isolation membrane^48,49^. The effects of CD24-dependent sorafenib resistance disappeared or diminished during the inhibition of autophagy using either pharmacological inhibitors or essential autophagy gene knockdown. Hence, CD24 regulates sorafenib resistance via autophagy activation. The AKT/mTOR pathway is an important upstream signal of autophagy activation^31^. However, this pathway was defective in sorafenib-resistant cells in a dose-dependent manner and was rescued by CD24 knockdown, suggesting that CD24-induced autophagy activation through inhibition of the AKT/mTOR pathway. Fujikuni et al.^50^ and other studies have shown that a hypoxic microenvironment can induce CD24 overexpression. Hypoxia inducible factor (HIF) can bind to the promoter of CD24, thereby promoting the invasion and metastasis of gastric cancer cells^50,51^. There are two-CD24 promoter sequences, the first from a pooled sample of human DNA^36^ while the second is from a population of B lymphocytes^37^. Factors may bind to the promoter of CD24 and recruit PP2A, which drives CD24 promoter activation and expression in sorafenib resistance. Protein PP2A, a multifunctional enzyme inhibitor of AKT activity, was significantly increased in Huh7/SR cells as seen in the RNA-Seq study.

Altogether, we found CD24 is highly expressed in sorafenib-resistant hepatocellular carcinoma tumor cells. CD24 was found to be a functional marker that was required for sorafenib resistance through AKT/mTOR mediated autophagy regulation. CD24 expression levels are also related to liver cancer progression and prognosis. All these findings provide potential therapeutic targets for HCC treatment.

## Materials and methods

### Patient samples and cell lines

A total of 70 paired HCC fresh tissues that consisted of tumors and adjacent normal samples were obtained from patients who underwent liver resection at the Liver Transplantation Center in The First Affiliate Hospital of Nanjing Medical University between October 2014 and November 2015. Thirteen of 70 patients underwent surgical resection after sorafenib treatment because of sorafenib resistance. Following approval by our Institutional Ethics Committee, all patients in our study offered their informed consent to take part in our study prior to surgery. All fresh tissues were collected and frozen in liquid nitrogen within 10 min. The diagnosis of all patients was histopathologically confirmed, and the clinical characteristics of all the patients are summarized in Supplementary Table 1.

The Huh7, Hep3B human hepatoma cell lines used in this study were obtained from KeyGen (Nanjing KeyGen Biotech Co., Ltd, Jiangsu, China). All the cell lines were cultured in DMEM medium (GIBCO, CA, USA) with 10% fetal bovine serum and 100 U/mL of penicillin sodium and 50 μg/mL strepomysin at 37 °C in humidified air containing 5% CO_2_. We established Huh7/SR resistant and Hep3B/SR resistant cells with long-term exposure to sorafenib (Selleckchem, Houston, USA) at low doses (0.625 μM) escalating to higher doses for a long period of time, up to a sorafenib concentration of 10 μM (the highest clinically achievable concentration), which the cell could survive.

### Quantitative real-time PCR

Total RNAs of fresh tissue samples and cells were extracted with TRIzol reagent according to the manufacturer’s instructions (Invitrogen, CA, USA). qRT-PCR was conducted to evaluate the expression level of the mRNAs of all relevant genes. GAPDH was used as the internal control, and all primers used are presented in Supplementary Table 2.

### RNA interference

Two shRNA sequences targeting CD24 and ATG5 were cloned into lentivirus vector GV248 (Gene, Shanghai, China) to knockdown CD24 and ATG5 expression, respectively, and the negative control shRNAs without sequence homology to human genes were provided by the same manufacturer. One microgram of gene-specific (shATG5, shCD24, primers used are presented in Supplementary Table 3.) or negative control vector was transfected into Huh7 cells in six-well plates at 80–90% confluence using Lipofectamine according to the manufacturer’s instructions. The CD24 and ATG5 knockdown cell lines were selected by incubation with media containing 1 μg/ml of puromycin.

### Cell proliferation and invasion assay

The proliferation ability of HCC cells was tested by the Cell Counting Kit-8 (Beyotime, Nantong, China) and EDU (5-ethynyl-2′-deoxyuridine) immunofluorescence staining assay (Millipore, MA, USA) according to the manufacturer’s instructions. Briefly, cells were incubated with 50 mM EDU for 6 h before fixation, permeabilization, and EDU staining, which were performed according to the manufacturer’s protocol. Cell nuclei were stained with DAPI (Sigma) at a concentration of 1 mg/mL for 20 min. The proportion of cells incorporating EDU was determined by fluorescence microscopy (Nikon, 80i, Japan).

### Immunohistochemical assay

The tissue samples were fixed in 4% paraformaldehyde at 4 °C and sectioned into slices. After deparaffinizing and rehydration, the sections were put into a pressure cooker for 5 min to restore the antigen in the nucleus using the citrate method. To reduce the background, H_2_O_2_ was used to suppress the endogenous peroxidase activity. The samples were blocked in normal goat serum with 5% BSA in TBS for 1 h at room temperature. The sections were incubated with primary antibody (1:400 dilutions) overnight at 4 °C and then washed with PBS three times. After incubation with secondary antibodies (16 h), the sections were subjected to a DAB reaction. The sections were photographed using a digitalized microscope camera (Nikon, Tokyo, Japan).

### Western blotting

To analyze the protein, tissue samples, and cultured cells were dissolved using a RIPA buffer (50 mM Tris, 1.0 mM EDTA, 150 mM NaCl, 0.1% Triton X-100, 1% sodium deoxycholate, 1 mM PMSF) (Beyotime, Nantong, China). Consistently, 30 μg of protein was loaded in each lane, fractionated by SDS-PAGE, and transferred onto a PVDF membrane. Then, the membrane was incubated at 4 °C overnight with human-specific phospho-mTOR(Ser2448) (Cell Signaling, 5536), mTOR (Cell Signaling, 2983), p62 (Cell Signaling, 8025), LC3B (Cell Signaling, 3868), ATG5 (Cell Signaling, 9980), PP2AA (Cell Signaling, 2039), PP2AB (Cell Signaling, 4953), CD24 (Abcam, ab76514), phospho-AKT (Thr308) (Cell Signaling, 13038), AKT (Cell Signaling, 4685), ABCG2 (Cell Signaling, 42078), (Abcam, London, UK), β-actin (Cell Signaling, 4970), and GAPDH (Cell Signaling, 5174) antibodies. The results were visualized by a chemiluminescent detection system (Pierce ECL substrate western blot detection system, Thermo Scientific, IL, USA).

### GENEWIZ NGS RNA-seq product report methods

Total RNA of each sample was extracted using a TRIzol Reagent (Invitrogen)/RNeasy Mini Kit (Qiagen)/other kits. Total RNA of each sample was quantified and qualified by the Agilent 2100 Bioanalyzer (Agilent Technologies, Palo Alto, CA, USA), NanoDrop (Thermo Fisher Scientific Inc.) and 1% agrose gel. Quantity of 1 μg total RNA with RIN value above 7 was used for the following library preparation. Next generation sequencing library preparations were constructed according to the manufacturer’s protocol (NEBNext® Ultra™ RNA Library Prep Kit for Illumina®). In brief, the PCR products were cleaned up using AxyPrep Mag PCR Clean-up (Axygen), validated using an Agilent 2100 Bioanalyzer (Agilent Technologies, Palo Alto, CA, USA), and quantified by Qubit 2.0 Fluorometer (Invitrogen, Carlsbad, CA, USA). Then libraries with different indices were multiplexed and loaded on an Illumina HiSeq instrument according to manufacturer’s instructions (Illumina, San Diego, CA, USA). Sequencing was carried out using a 2 × 150 bp paired-end (PE) configuration; image analysis and base calling were conducted by the HiSeq Control Software (HCS)+OLB+GAPipeline-1.6 (Illumina) on the HiSeq instrument. The sequences were processed and analyzed by GENEWIZ. The detailed information was uploaded to ArrayExpress with accession E-MTAB-6346.

### Electron microscopy

Cells were fixed with 2.5% glutaraldehyde with 0.1 M sodium cacodylate and stored at 4 °C until embedding. Samples were post-fixed with 1% osmium tetroxide, followed by an increasing dehydration gradient step using ethanol and propylene oxide. Samples were then embedded, and ultrathin (50–60 nm) sections were cut using an ultramicrotome (LKB-I). Images were examined with a JEM-1200 electron microscope at 80 kV after the samples were stained with 3% uranyl acetate and lead citrate.

### Xenotransplantation studies and histological analysis

Cells were suspended in 50% Matrigel and implanted subcutaneously into the dorsal flanks of 6-week-old female nu/nu mice. Tumor growth was monitored using digital calipers, and the volume was calculated using the formula: tumor volume (mm^3^) = [width (mm)] ^2^ × length (mm) × 0.5. Mice were subjected to an intraperitoneal injection of sorafenib (100 mg/kg; Selleckchem, Houston, USA), BafA1 (20 mg/kg; Selleckchem, Houston, USA), or dimethylsuifoxide (Selleckchem, Houston, USA) delivered via intratumoral injection twice weekly for 2 weeks. All xenograft experiments were completed at least two independent times. The sample size for the groups was projected based upon previous xenograft studies and adjusted following data acquisition in the initial experiment. Tumors were collected for histopathological analysis and flow cytometry. Animals were only excluded from analyses in the event of death from procedure-related causes (for example, sepsis, tumor size) that were unrelated to experimental differences between the groups.

### Statistical analyses

The data are presented as the means ± standard deviations (SD). Tukey’s test or Student’s *t*-test for the unpaired results was used to evaluate the differences among more than three groups or between two groups, respectively. Differences were considered significant for values of *p* < 0.05.

## Electronic supplementary material

Supplementary Table 1

Supplementary Table 2

Supplementary Table 3

Supplementary Figure 1

Supplementary Figure 2

Supplementary Figurelegend
