# Supplementary figures and images for: CD24 regulates sorafenib resistance via activating autophagy in hepatocellular carcinoma
Source: Cell Death Dis. 2018 May 29;9(6):646. doi: 10.1038/s41419-018-0681-z (PMC5974417; doi:10.1038/s41419-018-0681-z)

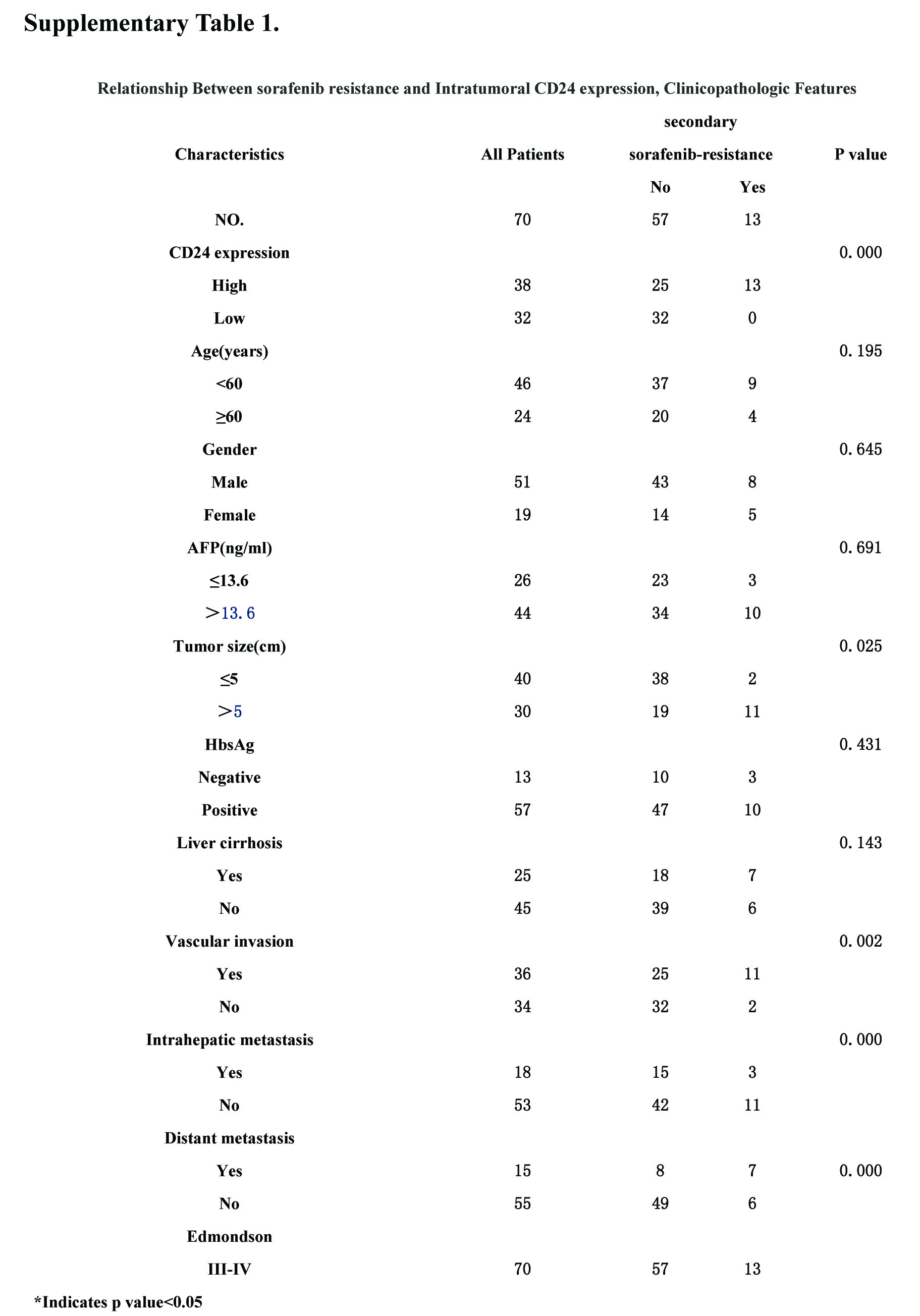

Supplement: Supplementary file 1 — Supplementary Table 1 [file 41419_2018_681_MOESM1_ESM.jpg]

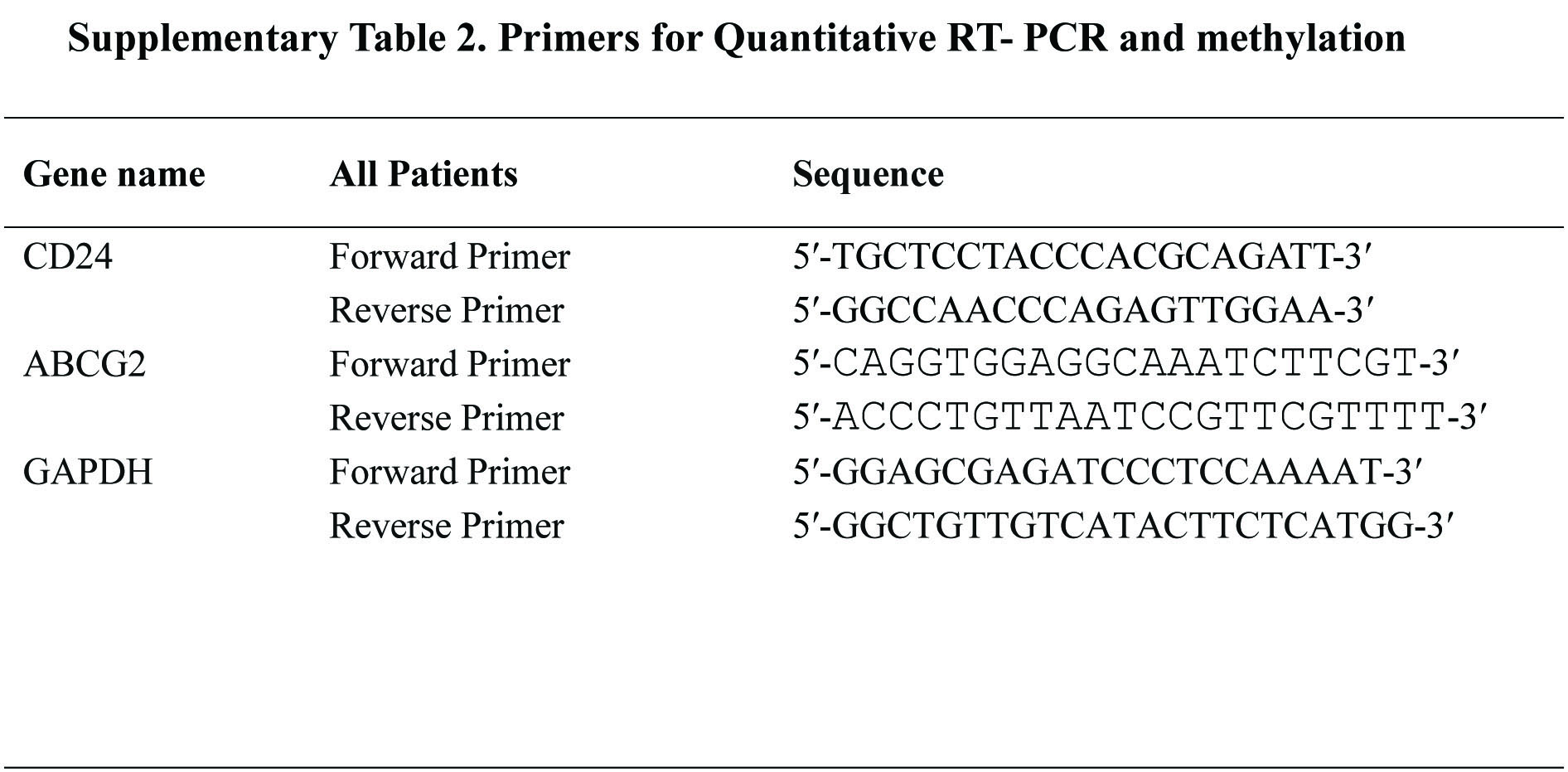

Supplement: Supplementary file 2 — Supplementary Table 2 [file 41419_2018_681_MOESM2_ESM.jpg]

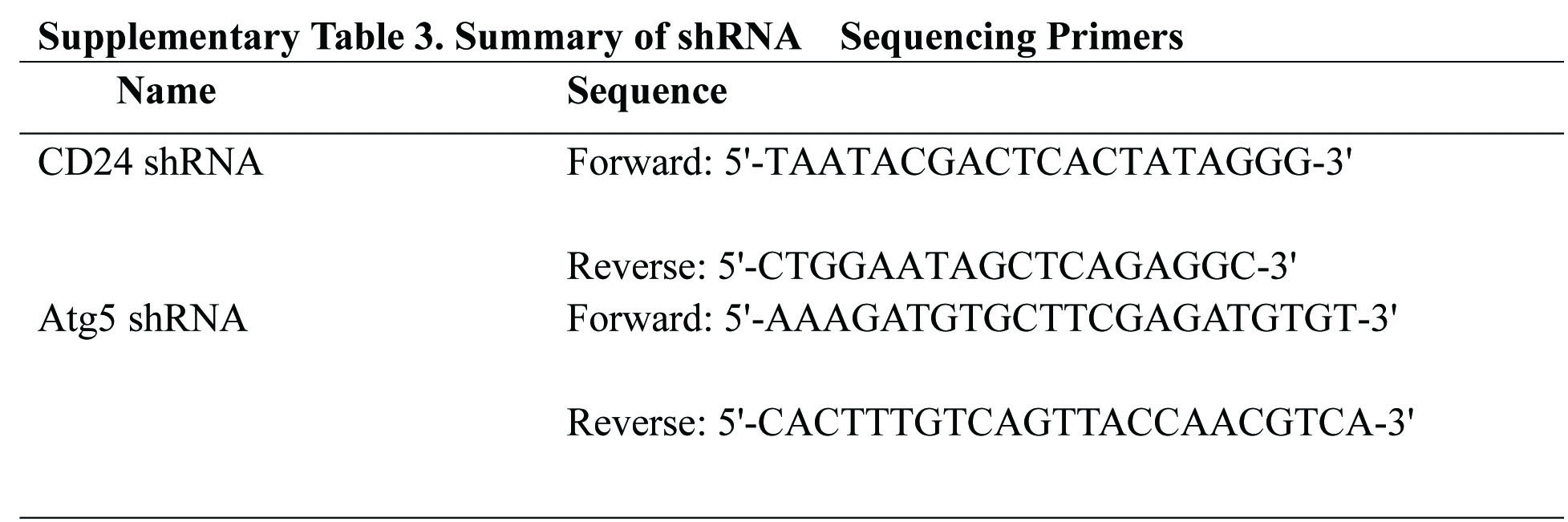

Supplement: Supplementary file 3 — Supplementary Table 3 [file 41419_2018_681_MOESM3_ESM.jpg]

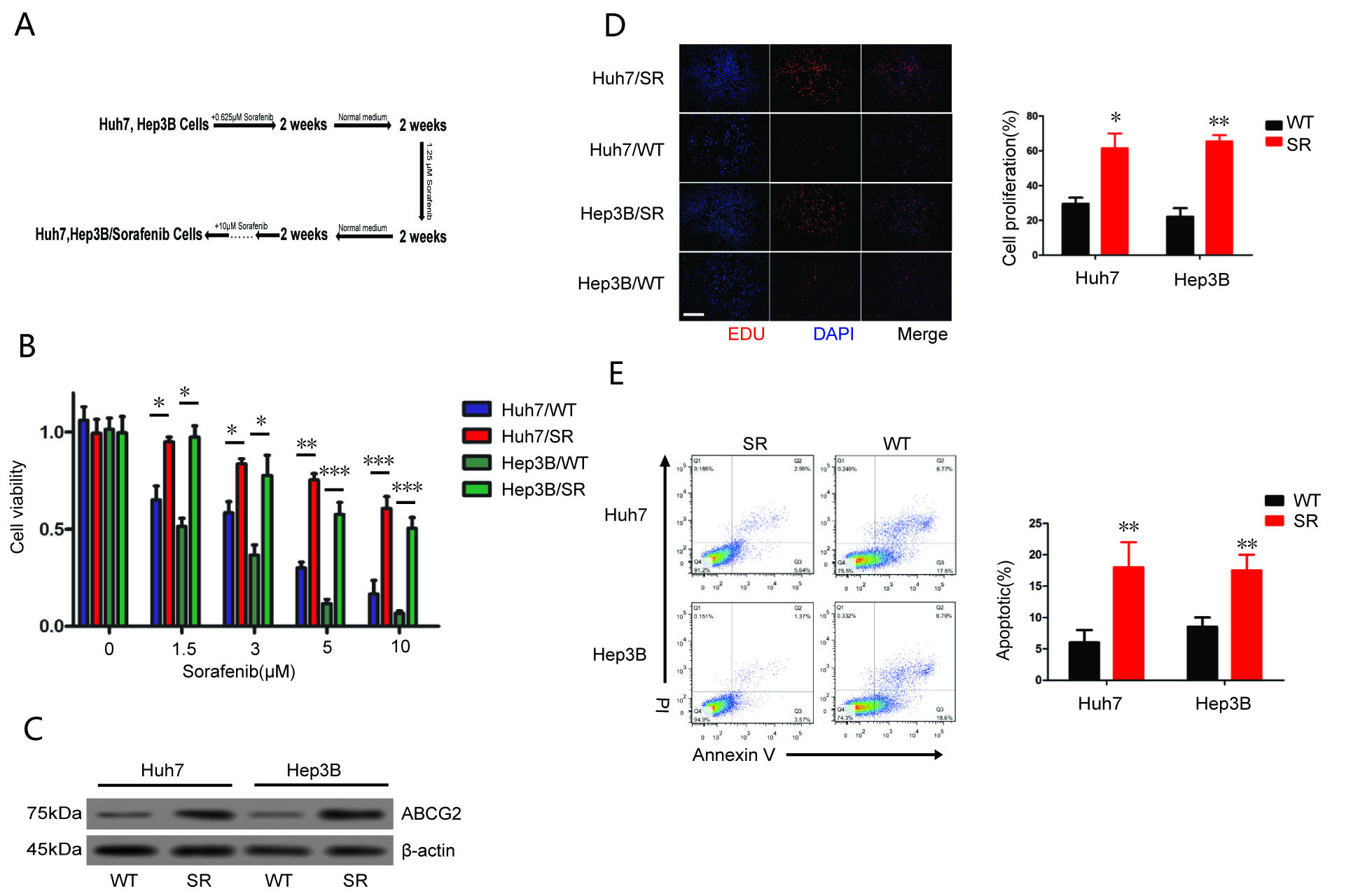

Supplement: Supplementary file 4 — Supplementary Figure 1 [file 41419_2018_681_MOESM4_ESM.jpg]

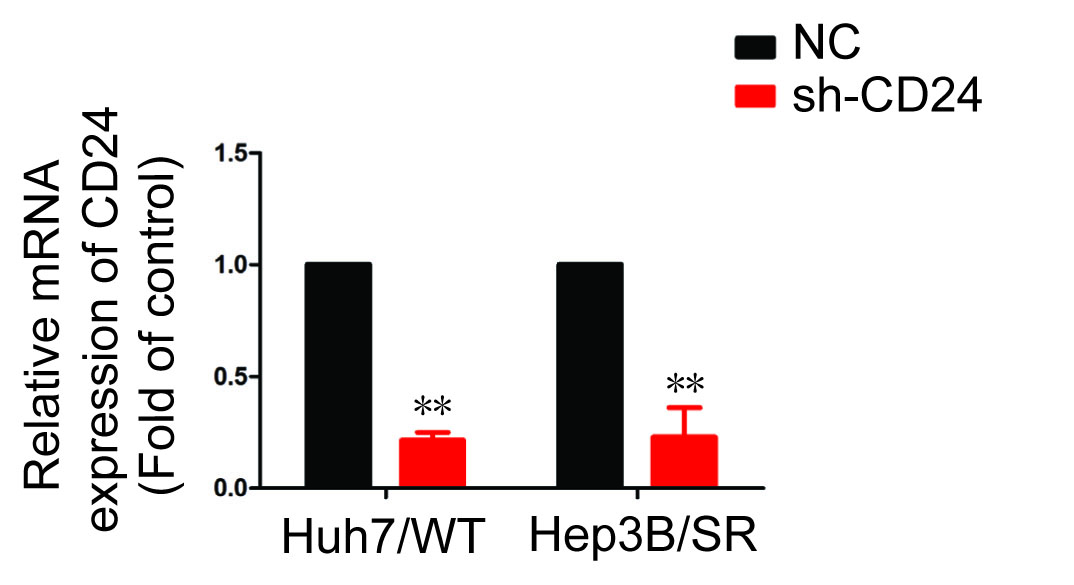

Supplement: Supplementary file 5 — Supplementary Figure 2 [file 41419_2018_681_MOESM5_ESM.jpg]
