# Supplementary material for: CD24 regulates sorafenib resistance via activating autophagy in hepatocellular carcinoma
Source: Cell Death Dis. 2018 May 29;9(6):646. doi: 10.1038/s41419-018-0681-z (PMC5974417; doi:10.1038/s41419-018-0681-z)
Supplement: Supplementary file 6 — Supplementary Figurelegend [file 41419_2018_681_MOESM6_ESM.doc]

***Supplementary Figure 1.*** ***Establishing a sorafenib-resistant HCC cell lines model***

As depicted in the diagram (A), we established several HCC cell lines by long-term exposure to sorafenib at low doses(0.625μM) escalating to higher doses for a long period of time, up to which the cell could survive in medium with 10μM (the highest clinically achievable concentration) sorafenib concentration. To understand the pharmacological and biological mechanisms of sorafenib resistance, (B) we measured sorafenib sensitivity via CCK8 assay (48 hours). (C) Western blot was used to detect the expression of ABCG2 protein. In addition, sorafenib-resistant cells exhibited higher (D)cell proliferation and (E)apoptosis in the presence of sorafenib(1.5μM) compared with wild-type cells. Representative images are shown on the left, and quantitative data are shown on the right. The original magnification was 200×. Each experiment was performed in triplicate. Data are presented as means±s.e.m. and analyzed with the Student t-test (*P<0.05, **P<0.01).

**Supplementary Figure 2**

QRT-PCR was used to assay the knockout efficiency of CD24. The experiment was peformed in triplicate. Data are presented as means±s.e.m. and analyzed with the Student t-test (*P<0.05, **P<0.01).
